# Different triggers for the two pulses of mass extinction across the Permian and Triassic boundary

**DOI:** 10.1038/s41598-021-86111-7

**Published:** 2021-03-23

**Authors:** Guoshan Li, Wei Liao, Sheng Li, Yongbiao Wang, Zhongping Lai

**Affiliations:** 1 Institute of Marine Sciences, Guangdong Provincial Key Laboratory of Marine Biotechnology, Shantou University, Shantou, 515063 China; 2 School of Earth Sciences, China University of Geosciences (Wuhan), Wuhan, 430074 China; 3 Anthropology Museum of Guangxi, Nanning, 530028 China; 4 No.3 Institute of Geological & Mineral Resources Survey of Henan Geological Bureau, Zhengzhou, 450000 China

**Keywords:** Geology, Palaeontology, Sedimentology

## Abstract

Widespread ocean anoxia has been proposed to cause biotic mass extinction across the Permian–Triassic (P–Tr) boundary. However, its temporal dynamics during this crisis period are unclear. The Liangfengya section in the South China Block contains continuous marine sedimentary and fossil records. Two pulses of biotic extinction and two mass extinction horizons (MEH 1 & 2) near the P–Tr boundary were identified and defined based on lithology and fossils from the section. The data showed that the two pulses of extinction have different environmental triggers. The first pulse occurred during the latest Permian, characterized by disappearance of algae, large foraminifers, and fusulinids. Approaching the MEH 1, multiple layers of volcanic clay and yellowish micritic limestone occurred, suggesting intense volcanic eruptions and terrigenous influx. The second pulse occurred in the earliest Triassic, characterized by opportunist-dominated communities of low diversity and high abundance, and resulted in a structural marine ecosystem change. The oxygen deficiency inferred by pyrite framboid data is associated with biotic declines above the MEH 2, suggesting that the anoxia plays an important role.

## Introduction

The Permian–Triassic (P–Tr) mass extinction^1^ (~ 252 Ma)^2^, destroyed both terrestrial and marine life^3^ and killed more than 90% of all species on Earth^1,4^. The extinction is the largest and most devastating biotic crisis of the Phanerozoic Aeon^5,6^ because it caused whole-scale reorganization in marine ecosystems and the transition from the Palaeozoic evolutionary fauna to the Modern evolutionary fauna^7^. While the exact causes and the nature of this extinction are unknown^6^, numerous hypotheses, including episodic volcanism^8,9^, rapid changes of sea level^10^, lethally hot temperatures^11^, widespread oceanic anoxia^12,13^, oceanic acidification^14^, oceanic toxicity^15^, and multiple catastrophic events^16^ have been proposed. Severe environmental perturbations related to giant volcanic eruptions have recently been considered one of the most likely killing mechanisms^9^. Oceanic anoxia across the P–Tr boundary is a global event and a major killing mechanism for extinction^17^. Besides, it explains the protracted recovery after the extinction^18–20^. However, the temporal dynamics of anoxia using proxies are still poorly constrained.

Various proxies such as sedimentological and palaeoecological features^12^, Th/U ratios^21^, cerium anomaly changes^22^, molybdenum isotopic composition^23^, uranium isotopes from dolostones^24^, sulfur isotopic excursions^25^, carbon isotopes^26^, and molecular biomarkers have been used to constrain the timing and nature of marine redox conditions during the crisis interval^27^. However, the geochemical proxies have several potential interpretations due to diagenesis^28^, and their applications are often limited in scope. The morphology and size distribution of pyrite framboids, which is a robust redox indicator found in both carbonate and mudstone/shale facies^29^, are a reliable proxy of benthic oxygenation in both modern environments^29^ and ancient sediments^30,31^.

In the South China Block, a small tectonic plate located in the eastern Tethys where the shallow-water platform and deep-water siliceous–argillaceous sedimentary facies coexist, pyrite framboids have been used as an indicator to investigate the P–Tr mass extinction. Numerous studies of pyrite framboid-inferred palaeoredox conditions have focused on deep ocean deposited sedimentary rocks^32,33^. However, a few have studied shallow platform sediments^34,35^, where microbialites are often bloomed as disaster forms after the mass extinction. Previous studies have suggested that a long-term and widespread oceanic anoxia occurred during the extinction^13^. Mounting evidence show that oceanic redox conditions underwent high-frequency oscillations^24,33,36^. Bond and Wignall^30^ suggested that anoxic conditions in the Palaeotethys were complex and unstable based on size distributions of pyrite framboids from several P–Tr boundary sections. However, those conodont data are not available in many Bond and Wignall sections^30^ to confirm the P–Tr boundary. Besides, the sampling density is also not high enough to reflect palaeoredox changes. Moreover, a depositional hiatus usually exists between conodont *Clakina meishanensis* and *Hindeodus changxingesis* zones in the shallow carbonate platform sediments in the South China Block^10^. Therefore, high-resolution palaeoecologic and sedimentologic studies from consecutive shallow settings are needed to reconstruct detailed oceanic palaeoredox changes during this critical interval.

Therefore, high-density and high-resolution samples were collected, and the triggers and pattern of mass extinction from the Liangfengya section were investigated. This study helps assess the temporal link between environmental triggers and two pluses of extinction by comparing it with the previous studies in the Yangtze Platform before, during, and after the mass extinction.

## Geological and stratigraphic settings

**Figure 1:**
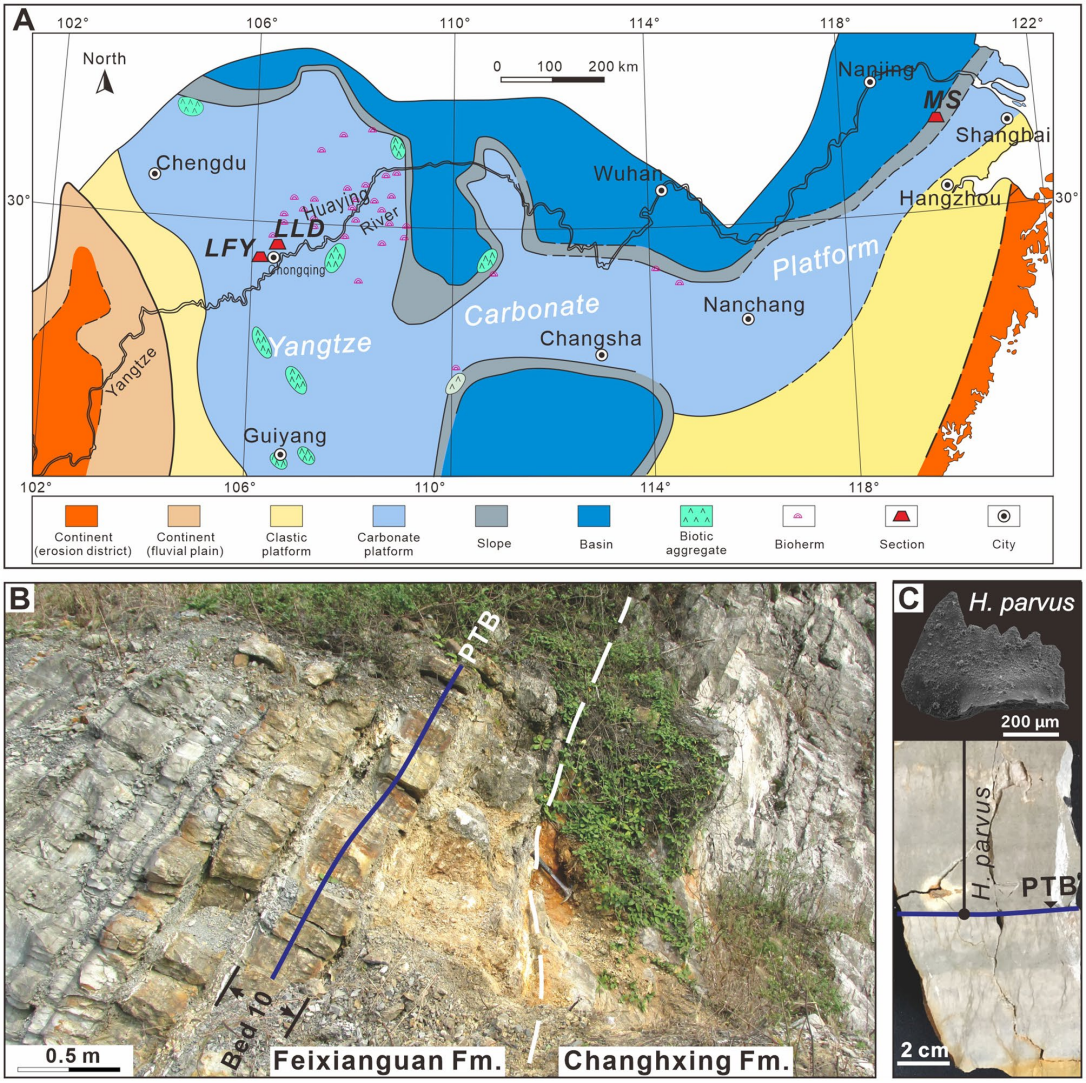
Location map and outcrop photograph of the Liangfengya section. (**A**) Late Permian palaeogeography of South China (modified from Feng et al.^44^) showing study section locations (*LFY* Liangfengya section) and previously-studied sections (*LLD* Laolongdong section^34^, *MS* Meishan Sect.^45^). (**B**) Outcrop photograph of the Liangfengya section showing the P–Tr boundary (PTB; solid blue line), and Changxing and Feixianguan Formation boundary (white dashed line). (**C**) Polished surface of Bed 10 from the Liangfengya section showing the first appearance of *Hindeodus parvus* and the P–Tr boundary (PTB; solid blue line).

**Figure 2:**
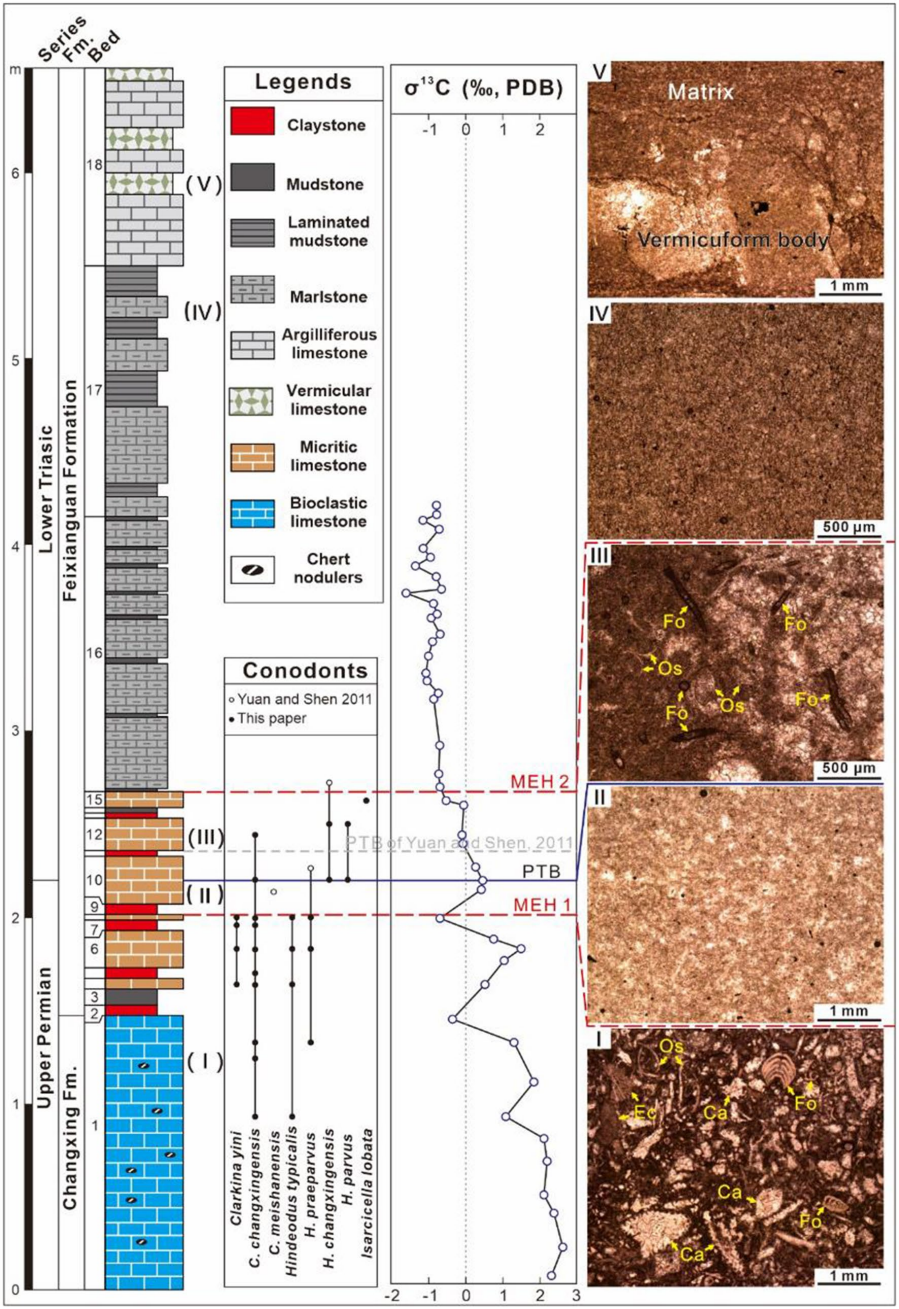
Stratigraphic log, conodont zones (open circles show Yuan and Shen data^38^), carbon isotopes, sedimentary microfacies, and fossil fragment assemblages of the P–Tr transitional sequence from the Liangfengya section. Photos include: (I) bioclastic limestone from the Changxing Formation, including abundant foraminifers (*Colaniella*, *Pachyphloia*), calcareous algae and ostracods; (II) micritic limestone from the Feixianguan Formation; (III) micrite from the Feixianguan Formation, including abundant *Earlandia* sp.; (IV) marlstone from the Feixianguan Formation; (V) vermicular limestone from the Feixianguan Formation. *MEH* mass extinction horizon, *PTB* P–Tr boundary, *Ca* calcareous algae, *Ec* echinoderm, *Fo* foraminifer, *Os* ostracoda.

Two different sedimentary facies coexisted in the South China Block (SCB) during the Changhsingian (Late Permian) [(1) shallow-water platform facies represented by various carbonate-dominated sediments and (2) deep-water siliceous/argillaceous deposits (Fig. 1)]. The Liangfengya section is located in an abandoned quarry in the Mt. Zhongliang area, 10 km west of Chongqing city, and the upper Yangtze Carbonate Platform (Fig. 1A). Yang et al.^37^ were the first to study it and since then it is one of the most important P–Tr boundary sections^38^. The Liangfengya area recorded a shallow-water carbonate platform, inter-reef facies deposits during the Late Permian^39^. There are representatives of Late Permian sponge-dominated reef facies in the northeast of Liangfengya^40^. This study analyzed the upper Changxing Formation and the basal Feixianguan Formation of the section (Fig. 1B). The total thickness of the section is about 6.4 m, with 18 beds (Beds 1–18) (Fig. 2). The topmost two meters of the upper Permian Changxing Formation, Bed 1, consists of medium to thick, bioclastic limestone, and yields abundant fossil fragments, such as foraminifers and fusulinids^41^, calcareous algae^42^, conodonts^38^, and calcareous sponges^40^. The lower part of the Feixianguan Formation is characterized by alternating yellowish shale and gray marlstones (Beds 2–15). The first episode of the mass extinction occurs at the topmost of Bed 8. It is characterized by the disappearance of a fossil assemblage dominated by fusulinids, calcareous algae^42^, and Permian brachiopods^43^. The basal five meters of the overlying earliest Triassic Feixianguan Formation comprise fine-grained lithologies including micrites and marls. Thin claystone, calcareous shales, and mudstone, sparse fauna separate the lithologies. Beds 9–15 contain a fossil assemblage with low diversity including small foraminifers, small gastropods, and ostracods. Beds 16–18 have a few bivalves, where pyrite framboids are common. Overall, three fossil assemblage groups suggest a two-pulses extinction event straddling the P–Tr boundary.

Conodonts are essential index fossils for P–Tr stratigraphic studies. Yuan and Shen^38^ established five conodont biozones within the Liangfengya section based on their occurrence. According to the first *Hindeodus parvus* appearance, the P–Tr boundary was placed at 81 cm above the bioclastic limestone of the Changxing Formation^38^. In this study, samples were collected at an interval of 5 ~ 10 cm from the upper Changxing Formation and lower Feixianguan Formation to conduct a high-resolution conodont stratigraphic study. Conodont *H. parvus* appeared in sample LFY 10-2 of Bed 10 (Fig. 1C), indicating that the P–Tr boundary is at the middle of Bed 10, located about 66 cm above the top of the bioclastic limestones of the Changxing Formation (Fig. 2), consistent with Peng and Tong result^46^.

**Figure 3:**
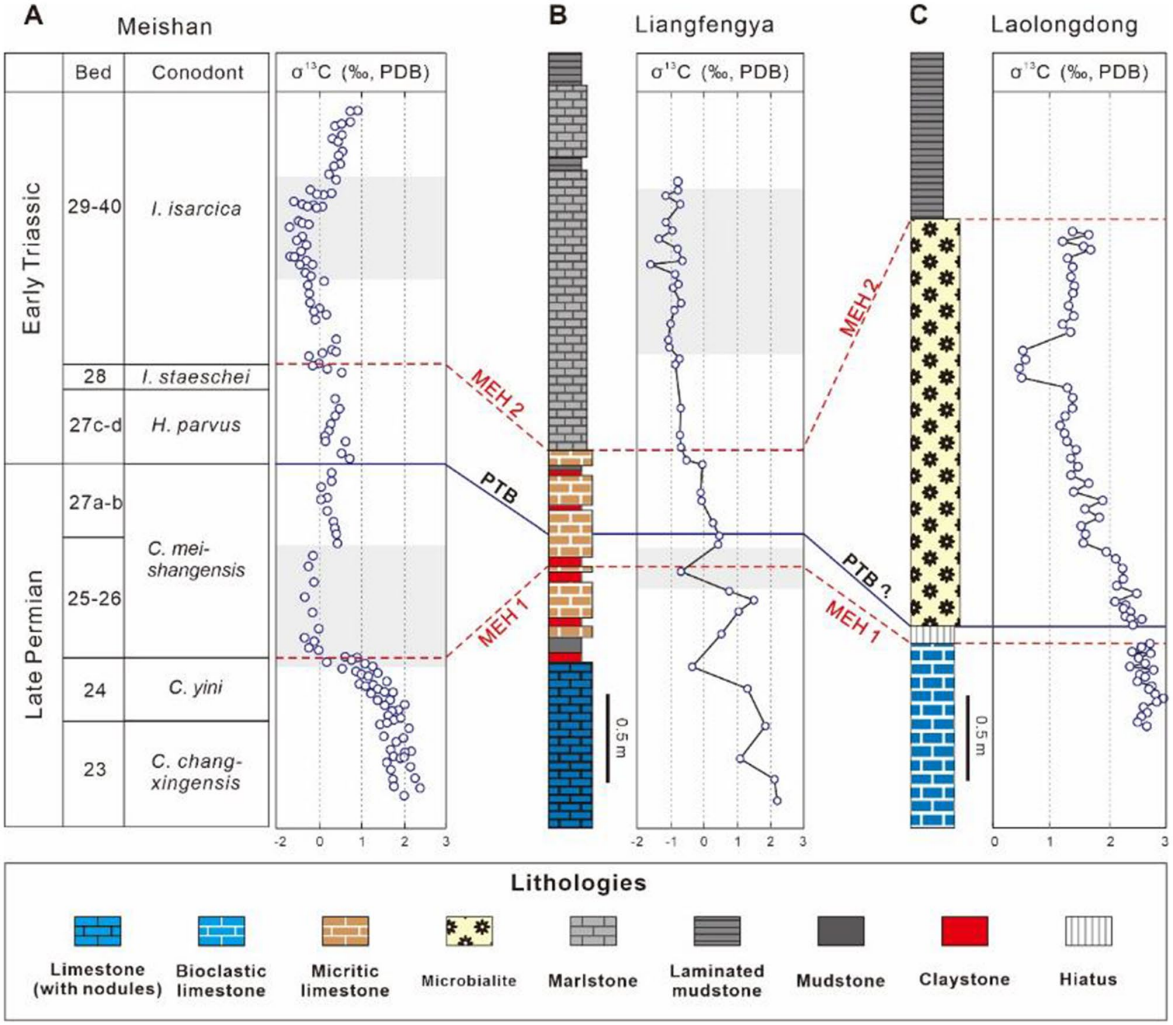
P–Tr boundary negative shifts correlation of δ^13^Ccarb in South China. Meishan from Xie et al.^45^; Laolongdong from Liao et al.^34^. The biostratigraphic frame of Meishan from Jiang et al.^49^. The biostratigraphic frame of Laolongdong revised from Yin et al.^10^. Shadowed areas represent negative excursions.

Besides conodont biostratigraphy, large carbon isotope excursions are useful for stratigraphic correlation from the end-Permian to the Early-Middle Triassic^47^. There are two negative excursions of δ^13^C_carb_ and δ^13^C_org_ across the P–Tr boundary in deep-water sections^26^. Two negative δ^13^C excursions occur at the Meishan section, within *Clarkina meishanensis* and *Isaricella isarcica* zones respectively, and are intercalated by a weaker positive shift around the *H. parvus* zone^45^ (Fig. 3A). However, due to the major regression at the end-Permian^10^, the first negative excursion record disappeared at most shallow-water sections (Fig. 3C). The δ^13^C_carb_ record from Liangfengya contains two negative shifts and a weaker positive shift, different from other shallow-water sections^34,48^ (Fig. 3), suggesting that there is no distinct hiatus within the Liangfengya section. The continuous sedimentary record of the Liangfengya section provides a potential to study triggers for the two pulses of mass extinction near the P–Tr boundary.

## Materials and methods

### Sample and fossil assembly

A total of 70 samples were continuously collected from fresh outcrops at the Liangfengya section, from a ~ 6.4 m thick interval across the P–Tr boundary. Changxing Formation had 11 samples, with a sampling interval of about 13 cm, and that of the Feixianguan Formation was about 7 cm.

A total of 29 samples were selected from the Liangfengya section to make directional thin-sections of 2.2 cm × 2.2 cm for fossil composition determination. Photomicrographs (41) were taken from the thin-sections and used to document the petrographic composition and taxonomic distribution of fossil fragments. A quantitative statistical method, with 300 or 500 points counted per thin section, was used to uncover the stratigraphic distribution of fossil fragments through the P–Tr transition^50^.

### Carbon isotope stratigraphy

Remarkable negative carbon-isotope excursions are an important characteristic of the P–Tr transition^26^. They are used for stratigraphic correlation from the end-Permian to the Early-Middle Triassic^47^. A total of 45 samples were selected for carbon isotope analysis to compare the P–Tr event at the Liangfengya section with other SCB sections. The weathered parts and diagenetic veins cutting across each sample were carefully removed during sampling. Finnigan-MAT 251 mass spectrometer was used to measure the isotopes. The results were presented in the conventional notation relative to the Vienna Pee Dee belemnite (V-PDB) standard. The analytical precision for δ^13^C was better than ± 0.1‰. The data showed one weaker positive and two significant negative excursions during the P–Tr transition in the Liangfengya section (Fig. 2) and were compared with the Meishan GSSP section^45^ (Fig. 3).

### Pyrite framboids analysis

In modern environments, aggregated iron monosulfides form at the redox interface between the oxic and anoxic zones^29^. In an anoxic/euxinic setting, pyrite framboids accumulate as tiny particles and have a narrow size range (i.e., small standard deviation). In contrast, framboids form at the redox interface within the surficial sediments when the lower portion of the water column is poorly oxygenated. The individual framboids are larger and more variable due to the local availability of reactants and the support of the sediment substrate (larger standard deviation)^29^.

Wignall and Newton^51^ demonstrated that morphology and size distributions of pyrite framboids are reliable indicators of palaeoredox conditions since five semi-quantify redox levels can be distinguished^30^. The pyrite framboid technique has recorded more instantaneous conditions^30^ and is valid for redox interpretations of weathered samples, different from many geochemical indicators^33^. Therefore, size analysis of pyrite framboids is a widely-used technique for evaluating the intensity and duration of ancient marine anoxic levels^33,36,51–53^.

In this study, 24 oriented thin-sections (ca. 2.0 cm × 2.0 cm) from across the P–Tr boundary were selected for detailed palaeoredox analysis. Two samples were from the bioclastic limestone of the Upper Permian Changxing Formation (Bed 1), five from the greyish-yellow micritic limestone of the P–Tr “transition beds” (Beds 2–15), and 17 from the Lower Triassic Feixianguan Formation (Beds 16–18). The samples covered the P–Tr transition interval recorded by this section, and the results reflected the palaeoredox evolution within the shallow carbonate platform of the upper Yangtze region. A stereomicroscope was used for preliminarily petrographic observation of polished block of each sample prepared and FEI Quanta 200 scanning electron microscope for the size of pyrite framboids in situ at the State Key Laboratory of Geological Processes and Mineral Resources, China University of Geosciences (Wuhan). At least 150 pyrite framboids size measurements were conducted for each sample where possible. Although such analyses appeared to underestimate the true size of the framboids when not measured from the great circle, calculation showed that the error had little effect on the interpretation of redox levels^29^. The pyrite framboid technique can be used for paleaoredox interpretations when the samples are weathered, unlike many geochemical proxies^33^.

## Results and discussions

### Paleaoredox changes in the Liangfengya section

Pyrite framboid distributions at the Liangfengya section were highly variable and underwent significant changes from the bottom to the top of the study interval. A total of 11 of 24 samples did not contain any framboids, but others yield pyrite framboids in variable concentrations. Generally, dark-colored thin mudstone/marlstone yields much smaller and richer framboids than light-colored thick limestone.

The lower part of the section (Beds 1 to 15), with bioclastic limestones of the Changxing Formation (Bed 1) and the micritic limestones of the lower part of the Feixianguan Formation (Beds 2 to 15) had seven samples. The samples had no pyrite framboids (only rare crystalline pyrite), suggesting that the bottom-water was oxic before and during the first extinction pulse. Other sections from shallow-water platform settings of the South China Block, including the Laolongdong section in Chongqing^34^, Gaohua section in Hunan^48^, and Panjiazhuang section in Zhejiang^54^, were also deposited under oxic conditions.

**Figure 4:**
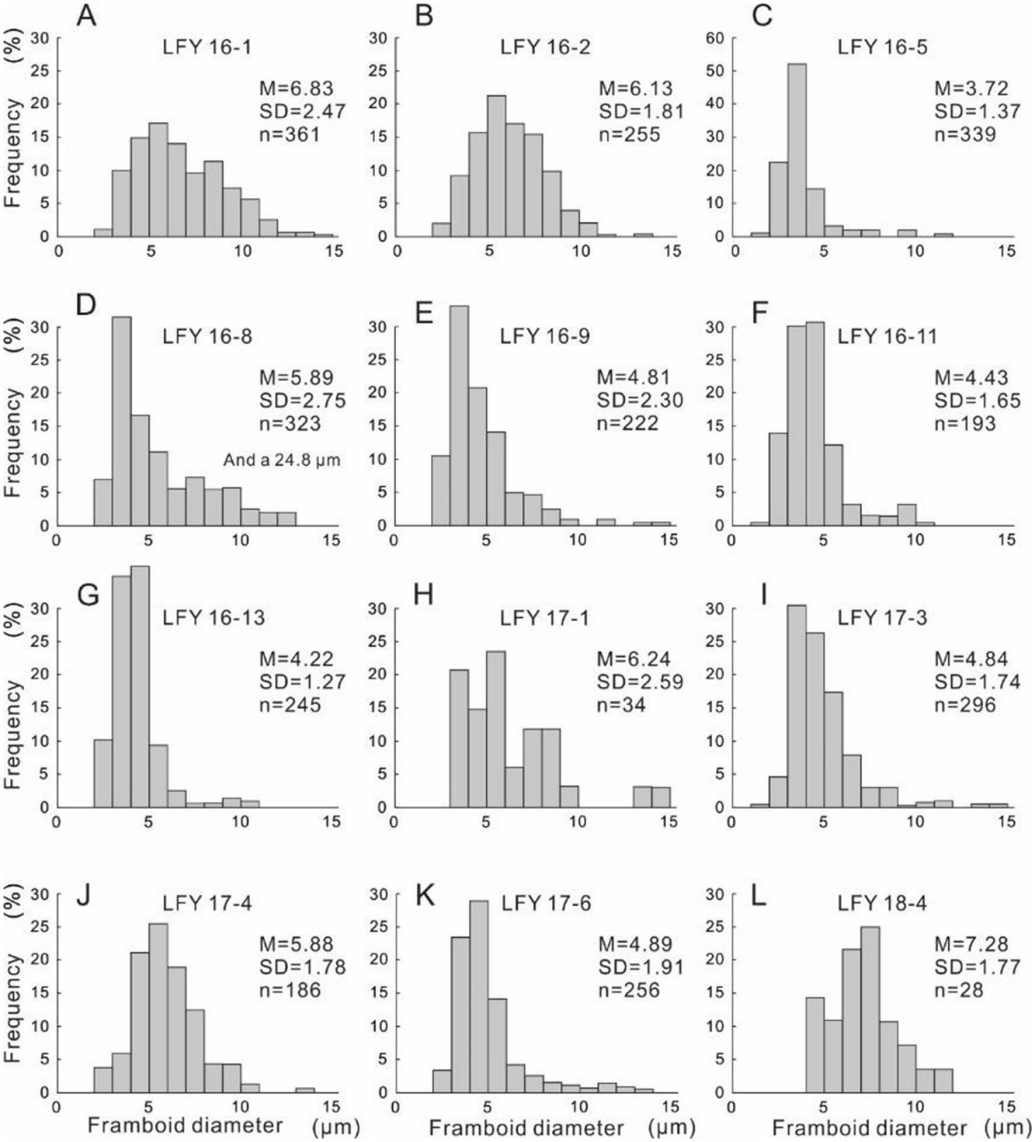
Size distributions of pyrite framboids from the Liangfengya section. Each histogram corresponds to samples marked from (**A**–**L**) in the stratigraphic section of Fig. 6. *M* mean framboid diameter (μm), *SD* standard deviation of framboid diameters (μm), *n* number of pyrite framboids measured in sample.

The marlstone and mudstone of the Feixianguan Formation (Beds 16 to 17), which lie above MEH 2, yield abundant pyrite framboids, with most dispersion in the matrix. Finally, the argilliferous and vermicular limestones of the Feixianguan Formation (Bed 18) had rare pyrite framboids, consistent with limited bioturbation observation in the samples. The size distributions of pyrite framboids from selected samples from Bed 16 to Bed 18 are shown in Fig. 4.

A total of 2738 pyrite framboids were measured from 12 samples (n = 186 to n = 361) except for LFY 17–1 and LFY 18–4, where only 34 and 28 framboids were identified, respectively. Generally, over 90% of pyrite framboids measured in each sample were smaller than 10 μm (Fig. 4). However, a few samples exhibited a “tail” of slightly larger framboids. Seven of 11 samples from Bed 16 had abundant framboids (about 69 per sq cm), with a mean framboid diameter between 3.72 μm (SD = 1.37 μm) and 6.83 μm (SD = 2.47 μm). However, four samples from the lower part did not contain framboids. Pyrite framboids from four samples in Bed 17 had similar size distributions to the upper part of Bed 16, which had a mean framboid diameter between 4.84 μm (SD = 1.91 μm) and 6.24 μm (SD = 2.59 μm). The abundance of measured framboids had a noticeable valley, about 9 per sq cm, at the bottom of Bed 17. In contrast, Bed 18, the vermicular and argillaceous limestones of the Feixianguan Formation, had rare framboids and some pyrite crystals. For instance, LFY 18-4 only had 28 pyrite framboids in the polished block, with a mean diameter (M) of 7.28 μm, and a standard deviation (SD) of 1.77 μm (Fig. 4L).

**Figure 5:**
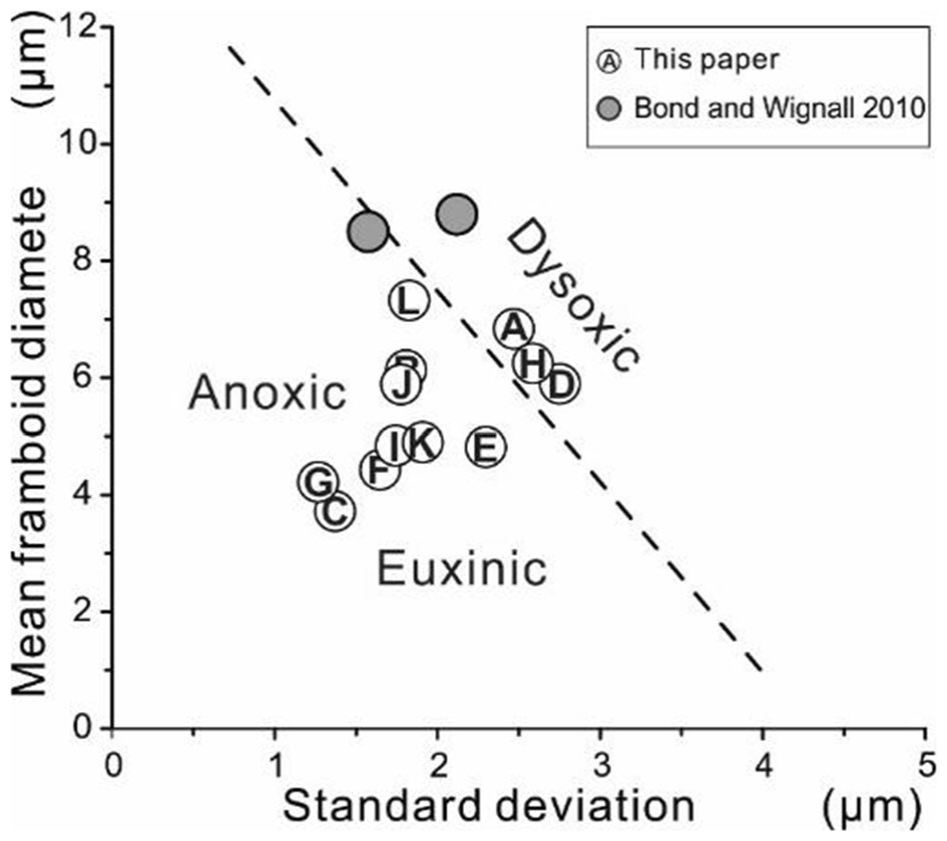
Mean diameter versus standard deviation crossplot of pyrite framboid data from the Liangfengya section. Each sample corresponds to those marked from (**A**–**L**) in the stratigraphic section in Fig. 6. The dotted line separating anoxic/euxinic from dysoxic facies is from Bond and Wignall^30^.

**Figure 6:**
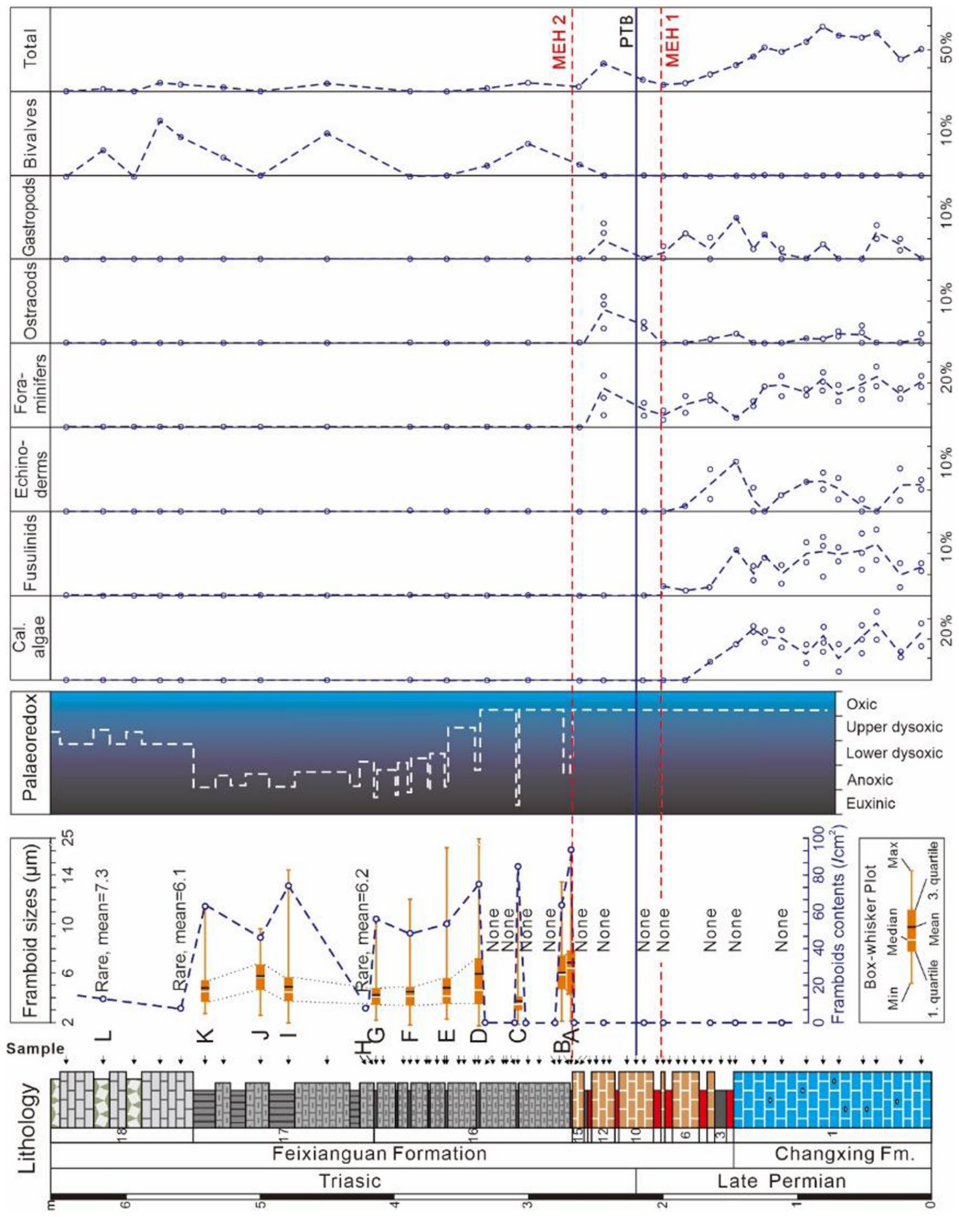
Pyrite framboid “box-and-whisker” plots of the Liangfengya section, the distribution of inferred water-column redox conditions according to Bond and Wignall^30^, and stratigraphic percentages of fossil fragments determined in thin-section. *MEH* mass extinction horizon, *PTB* P–Tr boundary.

Semi-quantify palaeaoredox levels were semi-quantified at the Liangfengya section using a cross plot of mean diameter versus standard deviation based on Wignall and Newton^51^ and Bond and Wignall^30^ criteria (Fig. 5). Five samples from Bed 16, three from Bed 17, and one from Bed 18 were within the anoxic/euxinic field, indicating poor-oxygen conditions in the water column (Fig. 5). Circulation was suppressed, and stratification reappeared in the water column during the rise in Early Triassic temperatures^11^. The absence of bioturbation in the samples determined via thin-section analysis supports the redox interpretation. There were two samples from Bed 16 and one from Bed 17 within the dysoxic field. “Box-and-whisker” plots showed the changing size distribution of pyrite framboids (Fig. 6), suggesting that palaeoredox condition of Liangfengya section varied between euxinic/anoxic to dysoxic immediately after the second extinction pulse.

There were few framboids (14 in sample LFY 18-1 and 28 in LFY 18-4) at the top of this section. However, the size distributions were along the edge of the anoxic zone (Fig. 5). According to the number and size of framboids, this stage was assigned to dysoxic conditions, consistent with limited bioturbation observed in vermicular limestone samples.

### Extinction pattern at the Liangfengya section

Two extinction horizons (MEH 1 & 2) straddling the P–Tr boundary at Liangfengya section were defined, as shown in Figs. 2 and 6. The benthic faunas underwent three significant changes: (1) the diversity and abundance of benthic organisms rapidly decreased, (2) algae and most fusulinids were extinct at the MEH 1 and, (3) bivalve abundance began to increase from the MEH 2 (Fig. 6). The total fossil abundance reached a peak (~ 71%) before MEH 1, and gradually decreased to ~ 8% in Bed 8. Abundance rebounded to 33% in Bed 12 after MEH 1, but substantially dropped in Bed 15, where it stabilized at or below 10% (Fig. 6). Notably, the calcareous algae suddenly disappeared at the beginning of the *Clarkina yini* zone^42^, during multiple layers of volcanic clay and yellowish micritic limestone (Beds 2–15).

Disaster forms, such as microforaminifers, ostracodes, and microgastropods bloomed within a short period in MEH 1 and MEH 2 (Fig. 6). Interestingly, the two distinct declines in abundance were associated with two microbial proliferations identified via biomarker analysis and negative shifts of carbon isotope composition^45^, possibly because foraminifers directly consume microbes, and their disappearance causes microbe blooming. Similar assemblage changes have been observed in many coeval faunas across the P–Tr boundary in sections from deep-water regions^47^.

Wu et al.^55^ proposed that mass extinction of reef ecosystems consists of two pulse and non-reef ecosystems similar to that of reef ecosystems. Song et al.^47^ demonstrated that the first stage of the extinction happens in the *Clarkina yini* zone and the second in *Isarcicella staeschei* zone. Song et al.^47^ used a “likelihood ratio” test on 87 species from the Liangfengya section, similar to the Meishan GSSP section. Their results confirmed a two-pulsed extinction pattern. A total of 62 species were consistent with a simultaneous extinction at the top of *Clarkina yini* zone^47^.

Liu et al.^41^ assumed that the foraminifers have a two-step decline at Liangfengya, similar to this study that showed a two-pulse extinction pattern in Liangfenya (Fig. 6), and Meishan^47^. However, according to conodonts biozones of Yuan and Shen^38^, Liu et al.^41^ pointed that the first extinction pulse was synchronous with Meishan, and the second within the *H. parvus* Zone. We confirm the two extinction pulses were synchronous at Liangfengya and Meiahan since conodont *H. parvus* was found in Bed 10.

### Different triggers for the two extinction pulses

**Figure 7:**
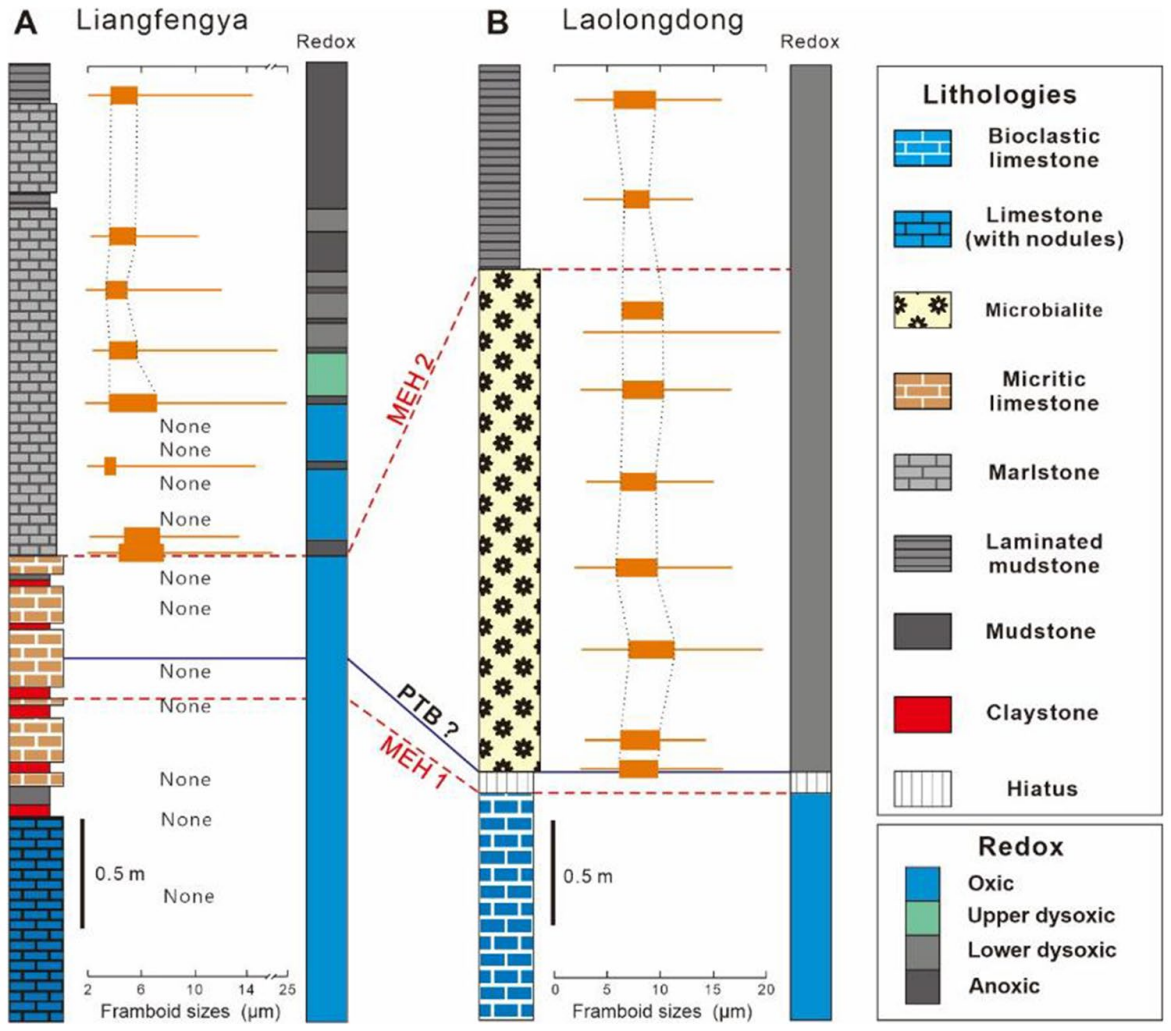
Stratigraphic logs of the Liangfengya and Laolongdong sections^34^, including pyrite framboid “box-and-whisker” plots and inferred redox conditions.

The study data showed a two-episode extinction pattern at the Liangfengya section. In contrast, many tropical shallow-water platforms only record the interlude phase between two major pulses of extinction^48^. There is no first extinction pulse record in most shallow platform sections because of the end-Permian regression^10^. Most normal marine benthos disappeared at the top of end-Permian reef limestone at Laolongdong section due to a hiatus of the end-Permian regression (Fig. 7)^34^. Post-extinction taxa, tolerant to anoxic or poor-oxygen conditions^55^, flourish at a low diversity in the overlying microbialite. The Liangfengya section data was good for the pattern or trigger comparison of the mass extinction during the P–Tr transition from shallow-water platforms to deep-water regions (Fig. 7). The group-specific extinction selection suggested that the two P–Tr extinction pulses have different environmental triggers.

Previous studies have shown a strong connection between the biotic mass extinction and oxygen deficiency in the water column^13,56^. For instance, Huang et al.^33^ found a two-stage oceanic anoxia pattern during the P–Tr transition in Kashmir, which is highly related to the two-pulse mass extinction^33^. Calcareous algae, fusulinids, and echinoderms disappeared at the top of Bed 8. None of the species were found in the P–Tr transition beds or the overlying Feixianguan Formation, as shown in Fig. 6. Sedimentary conditions are well-oxygenated according to the pyrite framboids of the Late Permian Changxing Formation. The shallow platform can support a diverse population under such conditions. However, Wignall and Hallam^39^ found that the marl (Beds 2–15) is poorly bioturbated, limited to small-sized burrows. Loope et al.^57^ reported a cerium anomaly from the microbialites at the Cili section in South China. They argued that shallow-marine anoxia was not widespread in equatorial waters and was not a factor in the mass extinction. Wu et al.^55^ pointed that non-reef ecosystems cannot experience oceanic anoxia but has the first extinction stage, implying that the oceanic anoxia is probably not the main killer of the oceanic benthos at the first extinction pulse.

Besides, there was a sharp temperature rise along with the P–Tr volcanism^11^. Chen et al.^58^ reported a ~ 9 °C increase in seawater surface temperatures from Bed 24e to Bed 27 at Meishan GSSP. High temperatures and increased terrigenous influx to the oceans during the Early Triassic could have increased the cyanobacterial bloom^45^, which constructed Lower Triassic microbialites in the SCB^34^ (Fig. 7). High and oscillating temperatures in the equatorial Paleotethys likely controlled the pace and nature of recovery after the end-Permian mass extinction^11^. Thus, high-temperature intolerant shallow-water inhabitants, such as corals and large foraminifers were first eliminated^11^, leading to calcareous algae loss. Taxa without physiological buffering and non-motile taxa were severely affected at higher extinction rates^59^. Ostracods and gastropods have high-temperature tolerances^16^, thus can survive in the shallow waters during the earliest Triassic. Therefore, the high temperature could be the main killer during the first extinction phase and not an anoxic condition. Besides, the end-Permian regression in *C. yini* Zone to *C. meishanensis* zone might have significantly affected the pattern and process of the PTB mass extinction^10^.

However, an anoxic event inferred from pyrite framboids occurred immediately after the MEH 2 of the Liangfengya section (Fig. 6). Similarly, the mean diameter of framboids in mudstone over the MEH 2 decreased at the Laolongdong section, suggesting a further intensification of oxygen deficiency in the bottom waters^34^ (Fig. 7). The oxygen-minimum zone (OMZ) expansion^60^ or upwelling of abyssal anoxic waters^61^ are used to explain anoxic condition development in shallow waters during the Early Triassic. Regional upwelling has been widely developed due to the particular palaeogeographical position of SCB^62^. Wignall et al.^63^ suggested that volcanic-induced global warming contributes to widespread oceanic anoxic event developments. The cyanobacterial bloom enhanced consumption of dissolved oxygen via degradation of organic matter produced by the cyanobacteria^31^. Furthermore, the carbon isotopes had a positive relationship with biodiversity (Figs. 2, 6). These results suggest that the change from oxic to dysoxic conditions in shallow environments could be related to the Early Triassic transgression^10,35^. Only a few planktonic or nektonic taxa such as epifaunal bivalves and conodonts can survive under anoxic conditions. The abundance and diversity of marine organisms were lowly maintained during the Early-Middle Triassic (Fig. 6). There were no disaster forms, such as microgastropods, ostracods, and foraminifers, flourishing in the microbialite before the MEH 2 in the lower interval of mudstone^34^. Moreover, deep-water slope facies (e.g. Shangsi section^64^), demonstrated a similar relation between the redox changes and the benthos extinction, suggesting that the anoxic conditions could have played an important role at the second extinction pulse.

## Conclusions

The Liangfengya section recorded a continuous shallow-water facies deposition from the Late Permian to Early Triassic. Besides, its biological assemblage revealed that the ancient halobios exhibited a two-pulse extinction pattern. The first episode of the extinction occurred during the latest Permian. The MEH 1 was at the top of the *Clarkina yini* zone (Bed 8), corresponding to Bed 25 of the Meishan section, characterized by the disappearance of all calcareous algae, echinoderms, fusulinids, and most large non-fusulinids foraminifers. The second extinction episode occurred in the earliest Triassic. The MEH 2 was at the top of Bed 15, corresponding to Bed 28 of the Meishan section. The second pulse selectively showed a different extinction. Some small foraminifers, ostracods, and microgastropods survived the first episode, but most were extinct during the second phase, causing a reorganization of marine ecosystems at the Liangfengya section.

This study showed two remarkable negative δ^13^C excursions and a weaker positive excursion near the P–Tr boundary correlated with the Meishan GSSP section. The two negative δ^13^C excursions, and the conodont biostratigraphy, indicate that there is no distinct hiatus within the Liangfengya section. The continuous sedimentary record provides a potential to study triggers for the two pulses of mass extinction near the P–Tr boundary.

The high-resolution analysis of pyrite framboid size distributions and sedimentary facies from the Liangfengya section provided a detailed shallow-water record of palaeoredox changes during the PTME. Notably, no framboid was found near MEH 1, suggesting that the first pulse of P–Tr mass extinction could not be caused by an anoxia event, at least for benthic organisms at Liangfengya. However, anoxic conditions inferred from framboid sizes immediately appeared above MEH 2, implying that anoxic conditions played an important role in the second pulse.

## Abbreviations

GSSP: Global stratotype section and point
MEH: Mass extinction horizon
PTB: Permian–Triassic boundary
PTME: Permian–Triassic mass extinction
SEM: Scanning electron microscope
SCB: South China Block
